# Biological Characterization of Microenvironments in a Hypersaline Cold Spring Mars Analog

**DOI:** 10.3389/fmicb.2017.02527

**Published:** 2017-12-22

**Authors:** Haley M. Sapers, Jennifer Ronholm, Isabelle Raymond-Bouchard, Raven Comrey, Gordon R. Osinski, Lyle G. Whyte

**Affiliations:** ^1^Centre for Planetary Science and Exploration, Faculty of Science, Western Science Centre, Western University, London, ON, Canada; ^2^Department of Earth Sciences, University of Western Ontario, London, ON, Canada; ^3^Department of Natural Resource Sciences, McGill University, Montreal, QC, Canada; ^4^Department of Food Science and Agricultural Chemistry, McGill University, Montreal, QC, Canada; ^5^Department of Animal Science, Faculty of Agricultural and Environmental Sciences, McGill University, Montreal, QC, Canada; ^6^Department of Physics and Astronomy, University of Western Ontario, London, ON, Canada

**Keywords:** cold spring, Mars analog, permafrost, phsycrophile, microbial ecology

## Abstract

While many habitable niches on Earth are characterized by permanently cold conditions, little is known about the spatial structure of seasonal communities and the importance of substrate-cell associations in terrestrial cyroenvironments. Here we use the 16S rRNA gene as a marker for genetic diversity to compare two visually distinct but spatially integrated surface microbial mats on Axel Heiberg Island, Canadian high arctic, proximal to a perennial saline spring. This is the first study to describe the bacterial diversity in microbial mats on Axel Heiberg Island. The hypersaline springs on Axel Heiberg represent a unique analog to putative subsurface aquifers on Mars. The Martian subsurface represents the longest-lived potentially habitable environment on Mars and a better understanding of the microbial communities on Earth that thrive in analog conditions will help direct future life detection missions. The microbial mats sampled on Axel Heiberg are only visible during the summer months in seasonal flood plains formed by melt water and run-off from the proximal spring. Targeted-amplicon sequencing revealed that not only does the bacterial composition of the two mat communities differ substantially from the sediment community of the proximal cold spring, but that the mat communities are distinct from any other microbial community in proximity to the Arctic springs studied to date. All samples are dominated by Gammaproteobacteria: Thiotichales is dominant within the spring samples while Alteromonadales comprises a significant component of the mat communities. The two mat samples differ in their Thiotichales:Alteromonadales ratio and contribution of Bacteroidetes to overall diversity. The red mats have a greater proportion of Alteromonadales and Bacteroidetes reads. The distinct bacterial composition of the mat bacterial communities suggests that the spring communities are not sourced from the surface, and that seasonal melt events create ephemerally habitable niches with distinct microbial communities in the Canadian high arctic. The finding that these surficial complex microbial communities exist in close proximity to perennial springs demonstrates the existence of a transiently habitable niche in an important Mars analog site.

## Introduction

Despite the dominance of permanently cold environments on Earth (Margesin and Miteva, 2011), the biological significance of the cryosphere has only begun to be recognized over the last decade (e.g., Priscu and Christner, 2004). Terrestrial cryoenvironments in polar regions are characterized by extremely low temperatures, limited water availability, and a thick permafrost layer. Diverse suites of cryophilic microorganisms have successfully colonized these environments and play key ecological roles in carbon and nutrient cycling (Margesin and Miteva, 2011). These extreme environments, characterized by low temperatures and often high salinity are exceptional analogs for putative habitable environments beyond Earth (Fairén et al., 2010; McKay et al., 2012).

The current atmospheric pressure on Mars precludes the formation of standing water at the surface. The presence of past liquid water is evidenced by the association of phyllosilicates with ancient crustal terrains, and subsurface liquid water interacted with the surface environment in catastrophic outflows during the Noachian (Lasue et al., 2013) to as recently as a few million years ago (Burr et al., 2002; Neukum et al., 2010). Liquid water on Mars, if it exists today, is likely in the form of subsurface eutectic brines in a spatially restricted hydrogeological cycle existing in thick permafrost (e.g., Martínez and Renno, 2013). Putative evidence of transient liquid water lies in observations of high-latitude seepage gullies (Malin and Edgett, 2000), recurring slope lineae (RSL) containing evidence of hydrated salts indicating briny water (Ojha et al., 2015) and somewhat more controversially, Martian slope streaks (Bhardwaj et al., 2017). The physiochemical parameters that characterize the cryoenvironments on Axel Heiberg Island, Canada, and specifically, the Gypsum Hill spring system represents a terrestrial analog for putatively habitable subsurface briny aquifers on Mars (Malin and Edgett, 2000; Andersen, 2002; Malin et al., 2006; Rossi et al., 2008; Davila et al., 2010; Fairén et al., 2010; McKay et al., 2012; Battler et al., 2013).

**FIGURE 1:**
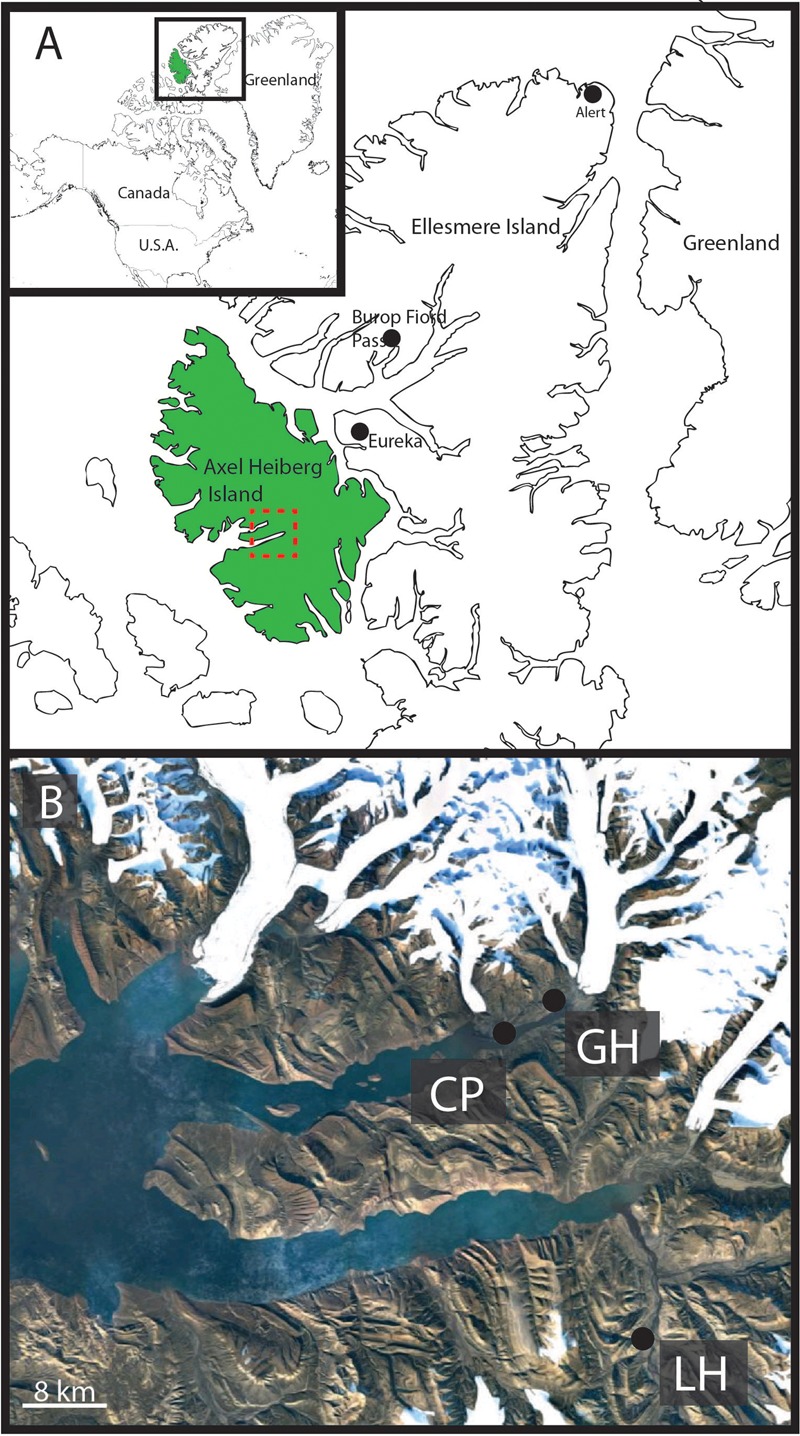
Geographic location of Gypsum Hill spring. **(A)** Axel Heiberg Island (green) and Ellesmere Island, boxed area shown in inset. Expedition Fiord, Axel Heiberg is outlined by the red box. Burop Fiord Pass, Ellesmere Island is indicated. **(B)** Satellite image of Expedition Fiord (red box, **A**). The three main hypersaline cold springs are indicated: LH: Lost Hammer, CP: Color Peak, GH: Gypsum Hill. Landsat-7 image courtesy of the U.S. Geological Survey.

Axel Heiberg Island, in the high Canadian Arctic hosts a series of perennial cold springs (Pollard et al., 1999; Omelon et al., 2001, 2006; Andersen, 2002; Pollard, 2005; Battler et al., 2013) (**Figure 1**) dominated by unique microbial communities (Grasby et al., 2004; Perreault et al., 2007, 2008; Steven et al., 2007; Niederberger et al., 2009, 2010; Lay et al., 2012, 2013; Lamarche-Gagnon et al., 2015) usually associated with deep subsurface or submarine environments. The hypersaline cold springs on Axel Heiberg Island are among the only known perennial springs flowing through thick permafrost on Earth (Andersen, 2002) and comprise a unique opportunity to study microbial diversity in cryoenvironments. Perennial spring activity in regions dominated by permafrost are extremely rare as the permanently frozen layer forms an aquitard effectively separating supra- and sub-permafrost aquifers limiting surficial activity to seasonal melt-associated events (Williams and van Everdingen, 1973). The extensive diapirism characterizing the Expedition Fiord area of Axel Heiberg Island creates a series of chemical tailks coupling supra and sub-permafrost reservoirs allowing for continual hypersaline fluid circulation and perennial spring activity (Pollard et al., 1999; Andersen, 2002; Harrison and Jackson, 2014).

The Gypsum Hill springs, one such system on Axel Heiberg Island, at nearly 80 N, is the only known nonvolcanic, hypersaline, sulphidic, perennial cold spring system on Earth. Detailed studies have characterized the microbial community of Gypsum Hill spring by assessing the diversity of the source sediments and water at four outlets (Perreault et al., 2007, 2008) and snow-covered run-off channels present in the winter (Niederberger et al., 2009). These studies indicate that the springs’ microbial community is primarily sustained by chemolithoautotrophic primary production performed by sulfur-oxidizing bacteria, with little to no evidence of phototrophic metabolism despite being surface exposed and in continuous illumination during the Arctic summer. To date, ecosystems of this type have been found only in permanently dark hydrothermal vents and sulfidic groundwater (Perreault et al., 2008). A study of streamers growing in snow-covered run-off channels forming during the winter months identified extremely limited microbial diversity again dominated by sulfur-oxidizing bacteria (Niederberger et al., 2009).

**FIGURE 2:**
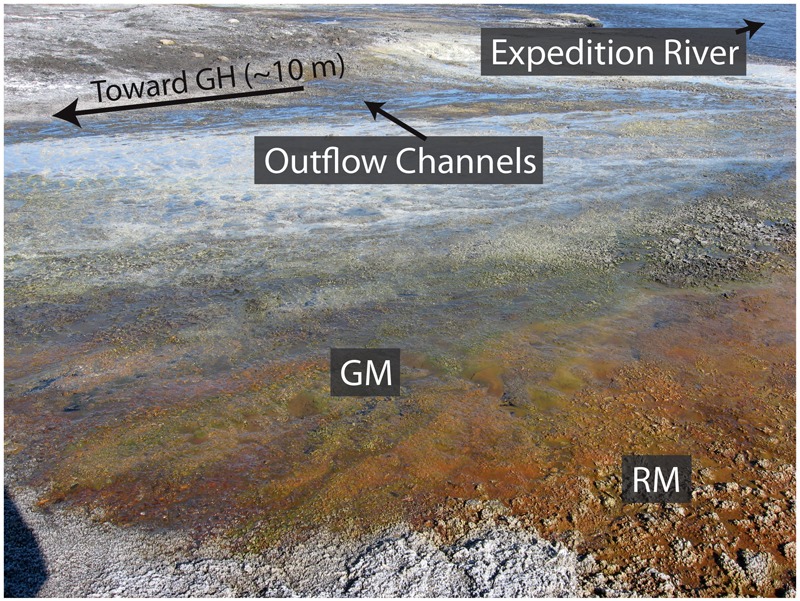
Visually distinct red and green mats forming the Gypsum Hill flood plain. The red (RM) and green (GM) microbial mats sampled in this study are shown relative to the Gypsum Hill (GH) outflow channels proximal to Expedition River. Note the intermixing of the microbial mats with the red mats dominating the areas furthest away from the GH outflow channels.

The microbial community and metabolic profile of surface waters from the Gypsum Hill springs suggests that the community may be derived from subsurface communities inoculated into the hypersaline fluids upwelling through the underlying Expedition diapir as opposed to seeding by surface-associated microorganisms (e.g., Perreault et al., 2008). Alternatively, the community may be evolved from the source water reservoir, having undergone selection during the residence time of the fluids in the subsurface. A previous study that identified *Marinobacter* within the Gypsum Hill springs suggested its presence may be due to a marine origin of the springs (Perreault et al., 2008). A flood plain hosting red and green microbial mats associated with the Gypsum Hill springs was observed during fieldwork in the summer of 2013 (**Figure 2**). An outstanding question is whether summer flood plains may support ephemeral, seasonal microbial communities colonizing the flood plain and how much diversity these seasonal communities share with the source pool. Such a question is important in understanding the role of perennial fluid flow in permafrost environments in sustaining and/or initiating microbial activity. Here we analyze the bacterial community in visually distinct mats in a proximal flood plain formed during the summer months and compare the results with the sediment-associated bacterial community in Gypsum Hill outlet 4 to assess the potential contribution of surface microorganisms to the spring community. While there are significant limits to using 16S rRNA targeted-amplicon sequencing data to assess microbial community structure, the data highlight compositional differences at high taxonomic levels and provide insight to plausible mechanisms of niche differentiation.

## Materials and Methods

### Gypsum Hill Spring

The Gypsum Hill spring system (79° 25′ N; 90° 45′ W) was originally described by (Beschel, 1963; Pollard et al., 1999) and is discussed in detail by a series of subsequent studies (Pollard et al., 1999; Omelon et al., 2001, 2006; Andersen, 2002; Battler et al., 2013). Gypsum Hill is one of at least 6 identified perennial saline spring systems on Axel Heiberg Island. Gypsum Hill is located in Expedition Fiord approximately 3 km from the White and Thompson Glaciers on the NE side of Expedition river. The system is comprised of 30–40 small outlets concentrated over an area of approximately 3000 m^2^ in a narrow band (∼300 m long × 30 m wide) that perennially discharge a combined average of 15–20 L s^-1^ of anoxic [mean oxido-reduction potential (ORP) ∼325 mV] brines (7.5–8% salts) at the base of Expedition Diapiar (Gypsum Hill). Outlet morphology is highly variable and seasonally dynamic, ranging from well-defined pools, to patches of brine-saturated sediment. Individual outlets discharge microaerophilic (0.05–0.2 ppm dissolved O_2_), hypersaline (8–9% salinity), and cold (-0.5–6.6°C) fluids at rates varying from <0.5–1.5 L s^-1^. The spring waters are near neutral pH (6.9–7.2) characterized by high concentrations of dissolved ions (dominated by Na^+^, Cl^-^, with lesser K^+^, Mg^2+^, Ca^2+^, SO_4_^2-^) and rich in both sulfate (2300–3724 mg^-1^) and sulfide (25–100 p.p.m.; Andersen, 2002; Pollard, 2005; Perreault et al., 2007). While the temperature of individual outlets vary (–0.5–6.6°C) between one another, fluid temperature (3 or 4°C average) is relatively constant despite significant seasonal changes in air temperature (Pollard, 2005). The physiochemical parameters of the springs have been detailed by others (Pollard et al., 1999; Omelon et al., 2001), and remain largely unchanged from the original chemistry reported by Beschel (1963). It is interesting, as first noted by Beschel (1963), that if all ions are normalized to Na^+^, the spring fluids are a chemical match to seawater. Although the physical and chemical characteristics of the fluid discharge have been well characterized, the water source is poorly constrained. It has been hypothesized that the springs originate from subpermafrost salt aquifers and rise to the surface through the permafrost (Andersen, 2002); however, the source aquifer has not been defined and the subsurface hydrogeologic system is largely unknown. An isotopic study by Pollard et al. (1999) found that the Gypsum Hill spring water did not deviate from the established Local Meteoric Water Line suggesting aquifer recharge under climatic conditions similar to modern. While Pollard et al. (1999) had originally presented five possibilities the most favorable including recharge from surface water bodies through tailks, ancient ground water circulation, and deep circulation of glacially pressurized water, Andersen (2002) developed a combined flow and thermal model to demonstrate that the brine discharge may originate in Phantom Lake, a glacially dammed lake several kilometers away.

### Field Work

Field work was conducted over two years in July 2013 and July 2014. During the summer of 2013 mats forming in a local flood plain characterized by local patchy color variation over a centimeter scale were identified proximal to outflow channels originating from Gypsum Hill outlet 4 (**Figure 2**). The microbial mats observed were 1–2 mm thick and underlain by dark-black silty water saturated sediments. While surface microbial mat communities are observed on a yearly basis during the summer research season (July–August, personal observations), the extent, appearance (color), range, and location of the mats can change seasonally. Over the course of three field seasons (2013–2015), we observed some mats in different locations, of different sizes, color, and appearance throughout the spring flood plain while other mat areas surrounding the predominant springs (i.e., high continuous flow with little changes over a 15 year observation period) were relatively consistent. During winter the springs become snow covered and it is likely that the flood plains, while not directly visible, are significantly disturbed if not absent. These observations suggest a seasonality and transient nature to the mat communities and a very limited duration of activity during the high Arctic summer months.

Areas dominated with green filamentous streamers (green mat; GM) were observed to be spatially associated with areas of red-brown sediment mats (red mat; RM). Samples of both GM and RM were collected July 10, 2013 by using a sterile 15 ml tube to scoop mat material beneath the air–water interface. Care was taken not to disturb or include the underlying sediment. Samples were frozen in the field and stored at -20°C until DNA extraction. During July 2014, two replicate sediment samples were collected from Gypsum Hill outlet 4 (GH-4, sediment A and sediment B). Sediment samples were frozen in the field and stored at -20°C until DNA extraction. Thus, a total of four samples were selected for downstream analysis, GH4-sediment A and GH4-sediment B from Gypsum Hill outlet 4 (GH-4) and green mats and red mats from the surficial flood plain.

### DNA Extraction, Sequencing, and Analysis

Total DNA was extracted from each sample using the RNA PowerSoil Total RNA Kit (MoBio, Carlsbad, CA, United States) and the RNA PowerSoil DNA Elution Accessory Kit (MoBio). Amplicons of partial 16S rRNA (bacteria) were produced for each sample using barcoded primers: forward 27Fmod (5′-AGRGTTTGATCMTGGCTCAG-3′) and reverse 519 (5′-TTACCGCGGCTGCTGGCAC-3′) targeting the V1–V3 hypervariable regions as in (McFrederick et al., 2013). High-throughput targeted-amplicon sequencing was performed by Mr. DNA (Molecular Research LP) (Shallowater, TX, United States) using the GS FLX+ Titanium platform (Roche, Branford, CT, United States) for the outflow channel samples (GM and RM). High-throughput targeted-amplicon sequencing was performed by Genome Quebec using the GS FLX+ Titanium platform for the sediment samples (GH-4).

Sequences were analyzed and classified using the suggested Mothur 454 protocol (Schloss et al., 2009). Briefly,.sff files were separated into.fasta and.qual files using ‘sff.info’ where flow = T. Sequence error was reduced as per Schloss et al. (2009) RM and GM sequences were trimmed using flowgrams with the ‘trim.flows’ command with the following parameters: order = A, pdiffs = 5, bdiffs = 2, maxhomop = 8, minflows = 450, maxflows = 450, while spring sediment samples were trimmed with the parameters: order = B, pdiffs = 5, bdiffs = 2, maxhomo = 8, minflows = 650, maxflows = 650. Sequence error was further reduced by ‘shhh.flows’. Sequences were reduced to only unique sequences using ‘unique.seqs’ and unique sequences that passed quality control for the sediment and mat samples were combined. Sequences were aligned to the Mothur-interpreted Silva database (version Silva.nr_v123) with the ‘align.seqs’ command. Aligned sequences were reduced to only the overlapping region using ‘screen.seqs’ and ‘filter.seqs’ (vertical = T, trump = .). Sequences were again reduced to only unique sequences using ‘unique.seqs’ then preclustered into OTUs consisting of two or fewer different base pairs using the ‘pre.cluster’ command (diffs = 2). Chimeras were removed using the uchime program within Mothur. A distance matrix was generated with ‘dist.seqs’ (cutoff = 0.15) and OTUs were formed based on this distance matrix using ‘cluster’. All sequences were classified using the Silva database mentioned above using ‘classify.seqs’.

A distance matrix generated of uncorrected pairwise distances between aligned sequences was constructed to compare the molecular diversity between samples. Distance matrices were generated for raw data and rarefied sample sets. The ‘count.groups’ command was used to determine that GH 4 spring GH4-sediment sample B had the lowest number of sequences, the ‘sub.sample’ was therefore used to rarefy all samples to only 4670 sequences. OTUs were classified using the ‘classify.otu’ followed by ‘phylotype’ and ‘make.shared’ so that a taxonomic comparison analysis could be completed. A representative sequence with the smallest maximum distance to the other sequences for each OTU was obtained and megaBLAST was used to find the closest uncultured and sequenced organism in the literature. Alpha diversity statistics were calculated on the OTU tables after rarefying all data sets to 4670 sequences.

Taxonomic stacked bar chart were rendered in R using the plot_bar function in phyloseq (McMurdie and Holmes, 2013). A heat map was constructed using the plot_heatmap function in phyloseq (McMurdie and Holmes, 2013) with method = NMDS and distance = bray.

### Scanning Electron Microscopy

Initial imaging of samples was conducted at the BioTron advanced imaging facility, Western University, London, Canada. Samples were lightly crushed using a sterile agate mortar and pestle and fragments transferred to carbon tape affixed to titanium TEM stub mounts and imaged at 40 Pa on a Hitachi 3400-N Variable Pressure Scanning Electron Microscope with an accelerating voltage of 10–15 kV at a working distance of 5–10 mm. High resolution imaging was conducted at the Western University Nanofabrication Facility. Samples were mechanically crushed using a mortar and pestle into ∼1 mm fragments under sterile conditions and fixed in 2% glutearalderhyde overnight at room temperature. Following fixation, samples were dehydrated in ethanol by subsequent 15-min immersions in increasing concentrations of ethanol (50, 75, and 100%) followed by a final submersion in 100% ethanol. The dehydrated samples were critical point dried with a Samdri-PVT-3B CP dryer (Tousimis Research Group) in the Biotron imaging facility to preserve cell structure. Samples were then manually transferred to carbon tape (M. E. Taylor Engineering part # DSCC-12) affixed to titanium TEM imaging stubs using a binocular microscope. Each sample was plasma coated with ∼10 nm of amorphous osmium using a Filgen OPC80T Osmium Plasma Coater with an OsO_4_ source. High-resolution imaging was carried out under high vacuum on a LEO Zeiss 1540XB FIB/SEM under an accelerating voltage of 1 kV and a working distance of 3.8 mm.

## Results

### Microbiology GM and RM

#### Molecular Variance

**FIGURE 3:**
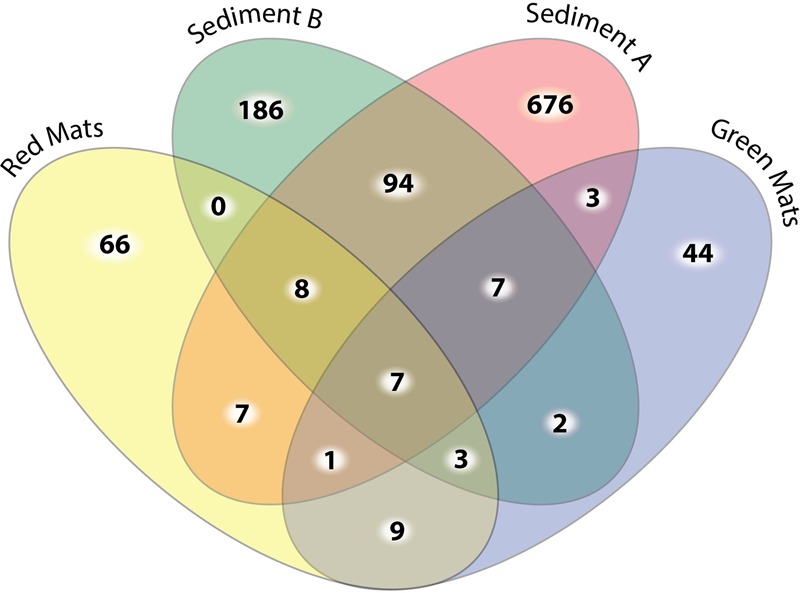
Four-way Venn diagram illustrating the number of shared unique OTUs at the 0.03 cut off level between samples. The sediment samples have the highest number of OTU diversity and the highest number of unique OTUs. Only 9 out of the 177 OTUs observed in the mat samples are observed in both mat communities.

To determine the microbial composition of the two mat communities standard alpha and beta diversity metrics were calculated. Following preprocessing of sequences, a total of 26,395 16S rRNA sequences were obtained and 1911 of these sequences were unique (**Figure 3**). The sequences were relatively evenly distributed between GH4 samples, GH-4sediment A contained 5849 sequences, GH4-sediment B 4670, green mats 7193, and red mats 8683.

#### Alpha Diversity

Alpha diversity indices were calculated to assess the diversity within each community. The 1911 unique sequences were assigned to 1113 OTUs with a cutoff value of 0.03. Coverage ranged from 91.7% (GH4-sediment A) to 99.4% (red mat). A higher number of OTUs were observed in the GH4 sediment samples (300–700) compared to the mat samples (60–80); however, measured diversity indices suggest that GH4-sediment A and the red mats had a higher diversity (inverse Simpson = 7.97 and 6.8, Shannon = 4.14, 2.42, respectively) than GH4-sediment B and green mats (inverse Simpson = 2.53, 3.33, Shannon = 2.31, 1.62 respectively; **Table 1**). This suggests that the OTUs accounting for the increased number of OTUs in the sediment samples might be rarely observed and account for a decreased evenness in these samples. This is supported by the high Chao1 values for the sediment samples (1392.7 and 814.5) as Chao1 will inflate species richness with the presence of rare OTUs. Shannon evenness varied from 0.39 (green mats) to 0.62 (GH4-sediment A) suggesting an uneven distribution of OTU abundance. Alpha diversity statistics are summarized in **Table 1**.

**Table 1:** Alpha diversity statistics for each sample randomly subsampled to 4760 sequences.

Sample	Coverage	Sobs	invSimpson	Shannon	Evenness	Chao
Sediment A	0.917509	803	7.966514	4.140907	0.630496	1393.740416
Sediment B	0.962527	307	2.530337	2.308351	0.403075	814.5
Green mats	0.993958	76	3.336399	1.623104	0.39232	103.678491
Red mats	0.994138	101	6.800028	2.424413	0.548936	119.404167

*Sobs = number of OTUs at a cutoff of 0.03.*

#### Beta Diversity

**FIGURE 4:**
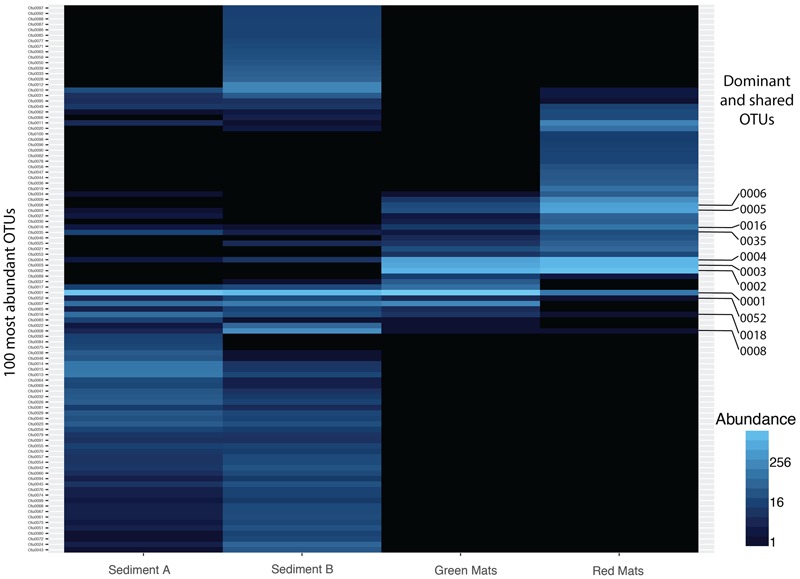
Heatmap illustrating the abundance of the top 50 OTUs across all samples. 7 OTUs are shared across all samples. Dominant (OTU 0001, 0002, 0003, 0004, 0005, 0006) and shared OTUs (0001, 0004, 0008, 0016, 0018, 0035, 0052) are labeled and presented in **Tables 2, 3**.

Beta diversity indices were used to assesses the variation in community structure between the red and green mats. A 4-way Venn diagram summarizes the distribution of the OTUs between samples (**Figure 3**) and the heat map presented in **Figure 4** illustrates major patterns in OUT membership between samples. The total OTU richness at a distance of 0.03 for all samples is 1113, with 803 OTUs observed in GH4-sediment A, 307 in GH4-sediment B, 76 in green mats, 101 in red mats. Only seven OTUs (0.6%) are shared between all samples. The greatest similarity is observed between the two sediment samples which share 94 OTUs or 11.7 and 30.6% of their total respective diversities. GH4-sediment A contains the highest number of unique OTUs (676), this is likely due to the high number of rare OTUs observed in this sample. Nine OTUs are shared only between the two mat samples.

**FIGURE 5:**
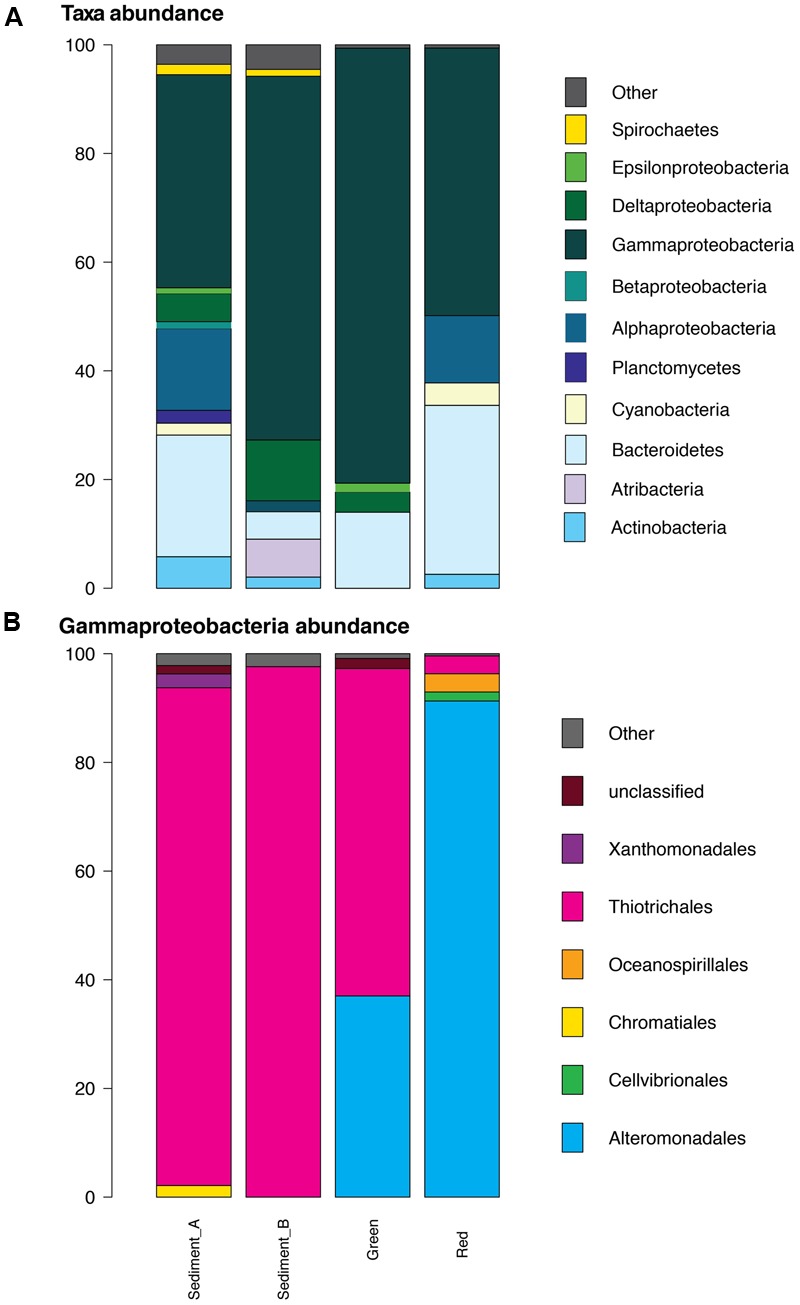
Bacterial composition of classified sequences at the >1% abundance level. **(A)** Phyla and Proteobacteria class. At the phylum level, all samples are dominated by Proteobacteria with the GH4 sediment samples having a higher relative diversity than the matt samples. **(B)** Gammaproteobacteria. Within the Gammaproteobacteria clade, Thiotrichales dominates the two GH4 sediment samples while Alteromonadales comprises a high proportion of the mat sample communities.

All samples were dominated by Gammaproteobacteria comprising 39, 67, 80, and 49% of each of GH4-sediment A and B, green mats and red mats, respectively (**Figure 5A**). Bacteriodetes is the next most abundant phylum composing 22, 5, 14, and 31% of each respective sample. The composition of Gammaproteobacteria varies between the two groups of samples (**Figure 5B**). The Gammaproteobacteria complement of the GH4 sediment samples is almost entirely composed of *Thiotrichales*, (92% and 98% GH4 sediment A and B, respectively) while green mats is 60% *Thiotrichales*, and 37% *Alteromondadales.* The red mats are almost exclusively *Alteromondales* (91%) with subordinate *Oceanosphillales* (3%) and *Thiotrichales* (3%).

#### Dominant OTUs

To better understand the dominant organisms in each community the most abundant OTUs are discussed. The seven OTUs shared between all samples account for over 40% of the total sequences. This core bacterial community is composed of Gammaproteobacteria (OTUs 0001, 0004, 0016, and 0052) assigned to the genera *Thiomicrospira, Marinobacter*, and *Rhodanobacter* (**Table 2**). The remaining shared OTUs are classified as Bacteroidia (OTU 0008, *Prolixibacter*), Epsilonproteobacteria (OTU 0035, *Sulfurospirillum*), and an unclassified Deltaproteobacteria (OTU 0018). The closest BLASTn representatives in the NCBI database are largely from marine, hypersaline, or psychrophilic environments. *Thiomicrospira* and *Sulfurospirillum* are associated with sulfur metabolism and are consistent with the known microbiology of Gypsum Hill (Perreault et al., 2007).

**Table 2:** Taxonomy of representative sequences from shared OTUs.

	Sample					Closest BLASTn representative			
OTU	Sediment A	Sediment B	Green	Mixed	Taxonomic classification (mothur, Silva V124 database)	Isolation location	*E*-value	Identities (%)	Acc number
0001	2004	2920	3464	143	Thiomicrospira	Sediment China: Hai River, northwest of Bohai Bay	8e-134	99	JF806846.1
						GH filament of sulfidic spring, Arctic	8e-130	100	EU430112.1
0004	8	2	700	1535	Marinobacter	Aquatic microbial mat from Antarctica	2e-135	100	FR772219.1
						Antarctic sandy intertidal sediments	2e-135	100	FJ889664.1
0008	335	4	1	1	Prolixibacter	Sediments from Rodas Beach polluted with crude oil	8e-89	90	JQ580434.1
						Guerrero Negro hypersaline microbial mat	8e-89	90	JN459039.1
0016	1	2	11	128	Marinobacter	Aerobic composting reactor	2e-125	98	HE647397.2
						Coastal marine sediment, Melville Harbor, Australia	8e-124	97	JQ032447.1
0018	25	92	8	1	Unclassified Deltaproteobacteria	Intertidal mudflatsediment vegetated by Sueada japonica in Ganghwa Island, Korea	4e-132	97	DQ112467.1
						carbonate-rich metalliferous sediment sample from the Rainbow vent field on the Mid-Atlantic Ridge	8e-129	96	AY354183.1
0035	3	17	2	25	Sulfurospirillum	Psychrophilic thiotrophic bacteria associated with cold seeps of the Barents Sea	2e-105	94	FR875418.1
						Psychrophilic thiotrophic bacteria associated with cold seeps of the Barents Sea	1e-102	93	FR875460.1
0052	22	4	5	1	Rhodanobacter	Shimokita Penninsula offshore drill core sample	1e-136	100	AB806773.1
						Shimokita Penninsula offshore drill core sample	1e-136	100	AB806772.1

OTU 0001 classified as *Thiomicrospira* is the dominant taxon in the GH4 sediment samples comprising 34 and 63% of the reads in the Sediment A and Sediment B samples respectively (GH4-sediment A: 2004 reads out of 5849 total sequences; 2920 GH4-sediment B: 2920 reads out of 4670 total sequences). The next most abundant OTU in GH4-sediment A is OTU 0008, Bacteroidia with 335 reads and in GH4-sediment B, OTU 0013, classified as a member of the class Desulfobacterales (**Table 3**). In contrast, the mat samples were not as clearly dominated by a single OTU. OTU 0001 is the most abundant OTU in the green mat with 3464 reads (7193 total sequences), but is only the eighth most abundant in the red mats (8683 total sequences). The most abundant OTU in the red mat sample is OTU 0002, classified as a *Marinobacter* species with 2227 reads. OTU 0002 is the second most abundant OTU in the green mat sample with 1448 reads. The second and third most abundant OTU in the red and green mat samples, respectively is 0003 classified as *Psychroflexus*, with 918 and 1568 reads.

**Table 3:** Taxonomy of representative sequences from dominant OTUs.

	Sample					Closest BLASTn representative			
OTU	Sediment A	Sediment B	Green	Mixed	Taxonomic classification (mothur, Silva V124 database)	Isolation location	*E*-value	Identities (%)	Acc number
0001	2004	2920	3464	143	Thiomicrospira (100)	Sediment China: Hai River, northwest of Bohai Bay	8e-134	99	JF806846.1
						Thiomicrospira psychrophila marine arctic sediments Svalbard: Store Jonsfjorden	2e-129	98	NR_042106.1
0002	0	0	1448	2227	Marinobacter (99)	subarctic glacial Fjord, Kongsfjorden	2e-135	100	JQ800125.1
						Marinobacter psychrophilus complete genome isolated from sea-ice of the Canadian Basin	4e-132	99	CP011494.1
0003	0	0	918	1568	Psychroflexus (100)	Tibetan Xiaochaidan Lake	1e-126	98	HM128393.1
						Psychroflexus sediminis haloalkaline soil Qinghai Province, northwest, Qaidam lake basin	1e-111	95	NR_044410.1
0004	8	2	700	1535	Marinobacter (100)	Aquatic microbial mat from Antarctica	2e-135	100	FR772219.1
						Marinobacter antarcticus Antarctic sandy intertidal sediments	2e-135	100	NR_108299.1
0005	0	1	27	787	Sulfitobacter (100)	Aquatic microbial mat from Antarctica	8e-119	100	FR772186.1
						Sulfitobacter japonica	2e-115	99	AB275995.1
0006	0	0	22	685	Psychroflexus (100)	Tibitan lake Xiaochaidan Lake	1e-122	97	HM128393.1
						Psychroflexus gondwanensis isolated from a defective cheese surface	1e-116	96	NR_117200.1

### Microbe–Substrate Interaction

**FIGURE 6:**
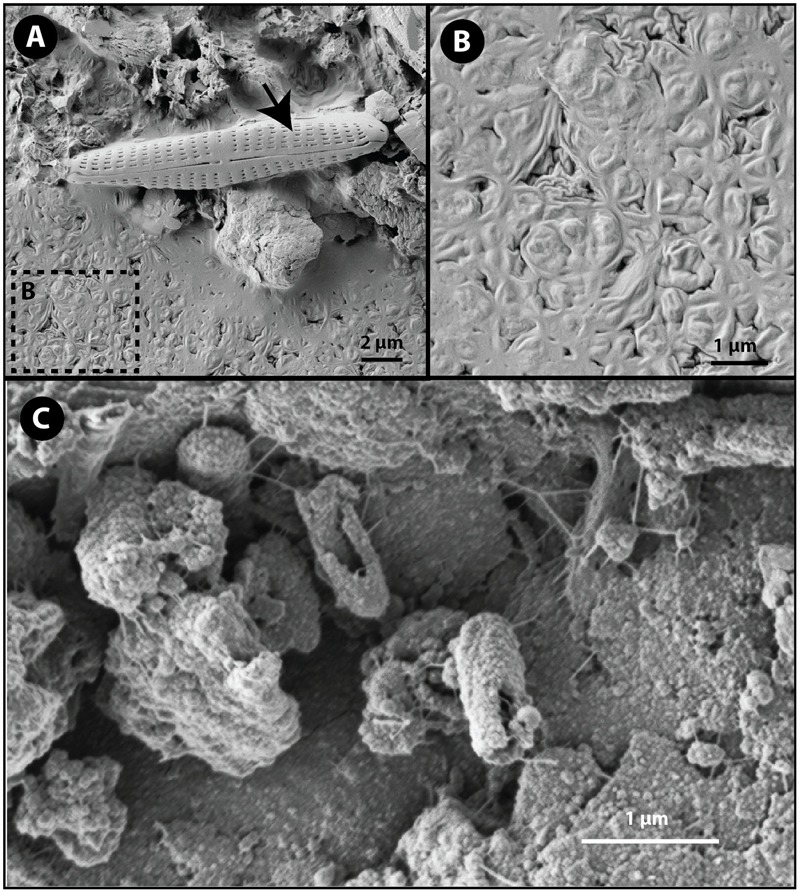
Secondary electron scanning electron microscope images of cell-substrate interaction. **(A)** Intact diatom (black arrow) and a desiccated coccoid biofilm (box B) dominated the GM samples. **(B)** The texture of the biofilm in GM samples is a result of desiccated and puckered coccoid cells. **(C)** Rod shaped cells adhered to the substrate in RM samples. Notice the rough surface textures of the cells consistent with biomineralization processes.

Secondary electron scanning electron microscopy (SEM) was used to evaluate cell-substrate associations in the mat samples. Imaging of GM samples revealed the presence of completely intact and undamaged diatoms as well as a massive biofilm (**Figures 6A,B**) while specific cell-substrate associations were not observed. As a result of limited samples and the inability to fix immediately in the field, the cells comprising the biofilm are misshapen and desiccated resulting in a puckered, non-canonical morphology despite attempts to preserve cellular morphology using standing fixation and critical point dying protocols prior to electron microscopy. Even though cellular morphology is not adequately preserved for morphological identification, electron microscopy did reveal differences between the red and green mat samples. The dominant bacterial taxon, *Thiomicrospira*, identified through 16S rRNA targeted-amplicon sequencing in the green mat sample is not known to form biofilms (Niederberger et al., 2009) and does not have a known coccoid morphology. The observed biofilm could be formed by algal cells. Algae were not targeted in the by the bacteria-specific primer sets used in the targeted-amplicon sequencing, and would also account for the green color characterizing the GM samples.

Scanning electron microscopy of RM samples revealed intimate associations between cocci and rod-shaped bacteria and the mineral substrate through filament-like extensions of extracellular substance (**Figure 6C**). The dominant rod-shaped cells are consistent with the morphology of *Marinobacter* and *Psychroflexus.* The rough surface texture apparent on the cells imaging in RM is suggestive of mineral precipitation (Ronholm et al., 2014). Cell-coating secondary mineral precipitation can occur through either passive or active biomineralization processes. A cultivation study of a supraglacial spring system Borup Fiord pass on Ellesmere Island, Canada (Gleeson et al., 2011) isolated a psychrophilic, sulfide-oxidizng consortia dominated by *Marinobacter* sp. that precipitated S (0). Subsequent dilution-to-extinction experiments indicated that *Marinobacter* sp. was the dominant sulfide oxidizer. While the majority of *Marinobactor* species are known for their ability to degrade hydrocarbons, the *Marinobacter sp* isolated by Gleeson et al. (2011) is the first known species to oxidize sulfur. This is consistent with a study conducted by Perreault et al. (2008) that identified *soxB*, a thiosulphate oxidation gene, in *Marinobacter* sp. *NP40.* Microscopy showed variably mineralized cells. The mineral coatings of the red mat (RM) cells may have preserved cellular morphology without the need for fixation techniques. The striking morphological difference between the RM and GM communities supports the differences in the genetic diversity as revealed by 16S rRNA targeted-amplicon sequencing. The observation of specific cell-substrate associations in the RM samples suggest that substrate-cell interactions play an important role in the RM communities, and the observation of putative mineral coatings in the RM cells suggests a role for biomineralization processes and perhaps lithoautotrophy involved sulfide oxidation.

## Discussion

### Flood Plain and Source Pool Bacterial Communities

There are substantial differences between the mat and GH spring sediment communities for classified OTUs at >1% abundance level within the Gammaproteobacteria clade. The Gypsum Hill spring sediment samples are dominated by *Thiotrichales* (>90%), similar to previous studies, while the samples from the mats have a significant *Alteromonadales* component (**Figure 5**). The community composition varies between the visually distinct mats suggesting spatially integrated, yet, biologically distinct bacterial communities. This division may reflect patchy geochemical variation, niche partitioning, or dynamic colonization as discussed in the following sections.

The core bacterial community shared between all samples is consistent with those found in studies comparing the bacterial composition of streamers forming in snow-covered run-off channels during the winter months to the source sediment of Gypsum Hill (Niederberger et al., 2009). The similarity in community structure between the streamers and Gypsum Hill source spring suggests a common source and it can be concluded that the bacterial complement in the runoff channels is, at least in part, derived from the spring outlet. In contrast, the core bacterial community between the mat samples and the source spring reported here comprises less than 1% of the total richness and there was a significant difference in the diversity between the two mat samples and between the mat samples and the source GH4 sediment. Given the significant difference in environment between the GH spring and flood plain, this difference in community structure is not surprising, however, supports the hypothesis that the community within the GH spring is not sourced from surficial microbial communities and is the surficial expression of deeper, subsurface community. We suggest that the seasonal outflow forming the flood plain provides an ephemeral habitat for transient surface microorganisms to colonize.

*Marinobacter* and *Psychroflexus*, the two most abundant OTUs in the red mat, and second and third most abundant in the green mat, respectively (**Figure 4** and **Table 3**), are not found in the GH4 spring sediment samples in this study, but have been identified previously from the Gypum Hill outlet (Perreault et al., 2008). In this study, the absence of *Marinobacter* and *Psychroflexus* in the GH sediments account for a large degree of the variation between the GH spring sediment samples and the mat samples. The absence of these phyla in the GH sediments in the present study may reflect the large degree of community variation. The top six OTUs, based on relative abundance, are summarized in **Table 3**. The dominant OTU in the green mat is 99% similar to *Marinobacter psychrophilus* at the 16S rRNA level, a nitrite reducer isolated from sea-ice in the Canadian basin (Zhang et al., 2008). The second most dominant, 95% similar to *Psychroflexus sediminis*, isolated from a hypersaline lake in China (Chen et al., 2009), is an obligate aerobic nitrate reducer. It is interesting to note that *Psychroflexus sediminis* produces non-diffusible orange carotenoid pigments (Chen et al., 2009) and this could explain the observable difference in coloration between the red and green mats. *Thiomicrospira*, the dominant taxon in the GH4 sediment samples, is an obligate chemolithoautotrophic sulfur oxidizer (Knittel et al., 2005). The dominance of *Marinobacter* as described here from the RM sample, has not been previously reported from the cold saline springs on AHI and suggests that in contrast to the outflow channels, the mat communities are not sourced directly from the spring. It would be pertinent to assess the microbial diversity in the active layer beneath the surficial mats to assess spatial variation in community structure with respect to changing physical and chemical conditions.

### Comparison to Other Microbial Communities

#### Hypersaline Microbial Mats

The spatial geometry of microbial mats and the physical coupling of diverse metabolisms, niche partitioning, and symbiosis allows mat communities to thrive in extreme environments. In contrast to established mat communities where conditions permit the lithification and growth of thick, cohesive microbial mats, the Gypsum Hill mats are patchy, discontinuous, only a few millimeters thick forming only on the surface of the permafrost active layer within flood plains and do not display any observable structural or community variation with depth. The physical structure of the Gypsum Hill mats make them difficult to compare with other hypersaline mat communities. Wong et al. (2016) reviewed the molecular ecology of 4 model hypersaline mat communities: Guerrero Negro, Shark Bay, S’ Avall, and Kiritimati Atoll. Similar to the Axel Heiberg mats, Proteobacteria were a major member in all four communities. Kiritimati Atoll was dominated by Bacteroidetes and Guerro Negro by Chloroflexi. Bacteroidetes contribution to overall diversity was, interestingly, also one of the major differences between the red and green mat communities studied here with the mats having a significantly higher proportion compared to the green mats. In Kiritimati Atoll, Bacteroidetes represented by *Salinibacter* and *Saprospiraceae*, were predominate in the surface layers contributing to photoheterotrophy (Schneider et al., 2013). In contrast, the main taxa contributing to Bacteroidetes in the red mat sample are *Psychroflexus* and *Gillisia.* Wong et al. (2016) suggest niche differentiation as an explanation for the emergent taxonomic structure variations within and between the four model saline mats and is consistent with our observations discussed in section “Hypersaline Microbial Mats”. Given the narrow vertical habitable range in the Gypsum Hill flood plain, the spatial organization of mat communities appear to be determined by lateral heterogeneity as compared to vertical stratification as observed in the other hypersaline mat communities.

#### Bacterial Diversity of AHI Springs

To assess the uniqueness of the mat communities on AHI, we attempted a systematic comparison of bacterial profiles from previous studies. We include datasets from previous studies of three perennial cold spring on AHI, Color Peak (CP), Gypsum Hill (GH), and Lost Hammer (LH), as well as data from a supraglacial spring in Borup Fjord pass on Ellesmere Island (BF).

Over the past two decades, a variety of techniques and approaches have been used to investigate the bacterial diversity of the perennial cold springs at AHI. These studies highlight the advances in microbial ecology: the earliest studies relying on <100 clones while in the last couple of years next generation sequencing technology has allowed for the analysis of tens of 1000s sequences and curation of 1000s of OTUs in a single study. The degree of bias between cloning and sequencing studies and NextGen amplicon sequencing and the increasing amount of information available in reference databases used for classification leads to great difficulty comparing studies. Three perennial cold springs on Axel Heiberg Island, Color Peak (CP), Gypsum Hill (GH), and Lost Hammer (LH), have been studied extensively with respect to their microbial communities. In addition to diversity studies of the main outlets of each spring, surficial run-off channels or outflow from GH and LH have also been examined. To date the bacterial communities characterized have been dominated by members of *Gammaproteobacteria* and *Bacteroidetes* known to be involved in sulfur cycling. Evidence for methanogenesis including the identification of several phylotypes consistent with methanogenesis and anaerobic methane oxidation were identified from LH. Overall the bacterial communities detected to date in the AHI spring systems are consistent with those identified and characterized from psychrophilic Arctic and Antarctic environments such as Antarctic sea ice and quartz sandstone (Nichols et al., 2005), Arctic and Antarctic pack-ice (Brinkmeyer et al., 2003), Arctic sea-ice (Zhang et al., 2008), including representatives from the phylotypes *Halomonas, Gillisia*, and *Marinobacter*.

**FIGURE 7:**
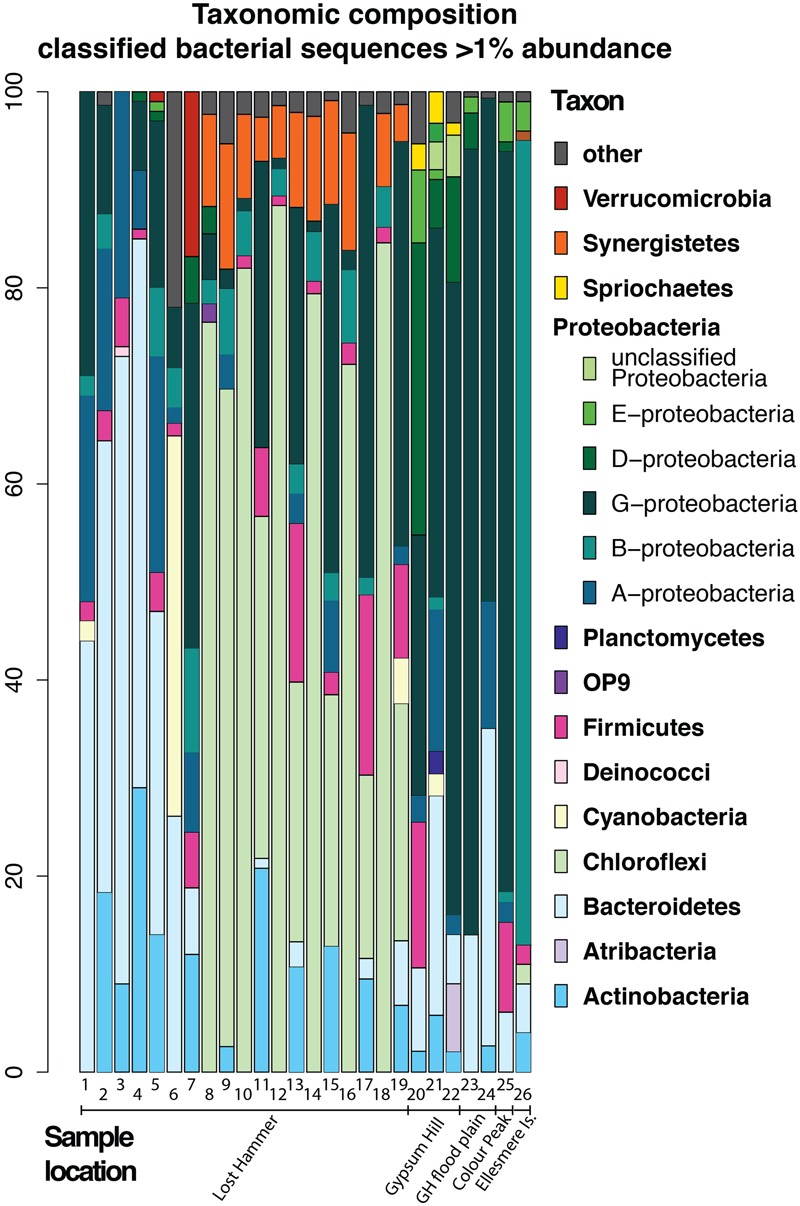
Bacterial composition of high Arctic communities of classified bacterial sequences present at the >1% abundance level. Samples 1, 6–19: Lost Hammer spring outlet, 2–5; Lost Hammer spring outflow, 20–22, Gypsum Hill spring outlet, 23–24 Gypsum Hill (GH) flood plain green mats and red mats, respectively), 25, Color Peak spring, 26, Borup Fjord, Ellesmere Island. Note the predominance of Chloroflexi in Lost Hammer spring samples 8–19, all from one study.

In an attempt to summarize the available information and investigate the most abundant phyla we have recalculated the reported bacterial abundances in each sample in several studies to create a dataset of 26 samples (Supplementary Table S1), each representing bacterial sequences classified at the phylum level, where each phylum is present at >1% abundance. It is important to note that the dynamic nature of reference databases may affect the classification of some OTUs, particularly for older studies. Given that adequate sampling depth requires the generation of thousands of clones and the largest clone library constructed for the AHI spring consisted of 161 clones, it can be assumed that these early studies were biased to detecting only the most abundant species. Therefore, we restrict our comparisons to the phyla level that comprise >1% of the community (**Figure 7**).

The issues correlating bacterial diversity results between different methods are apparent in the studies of Lost Hammer (LH) Spring (Niederberger et al., 2010; Lay et al., 2012; Lamarche-Gagnon et al., 2015). The distinction between the cDNA and DNA libraries found in both the cloning and sequencing (Niederberger et al., 2010; Lay et al., 2012) and amplicon studies (Lamarche-Gagnon et al., 2015) suggests a background bacterial population not necessarily related to the active community. However, the results of the pyrosequencing study (Lamarche-Gagnon et al., 2015) contrast sharply with the previous data indicating a predominance of Choroflexi, a phyla not previously observed. If all studies are biased to the most abundant phyla, the DNA studies may be recording variation in the dormant/dead population distinguishable over a longer time frame than the single seasonality studies (Lamarche-Gagnon et al., 2015) as high ionic strength, low temperature fluids increase the long-term stability of DNA (e.g., Lay et al., 2012). The differences may also be a result of changes to reference databases. The availability and quality of the sequence data precluded reclassification using a standard modern database. It is also important to note that sequences represent DNA [16S rRNA gene clone libraries: Niederberger et al., 2010 (LH), Lay et al., 2012 (LH), Perreault et al., 2007 (GH), Perreault et al., 2007 (CP)], 16S rRNA pyrosequencing: [Lamarche-Gagnon et al., 2015 (LH), Gleeson et al., 2011 (BF), this study (GH), cDNA (Lay et al., 2013 (LH), and metagenomic data (Lay et al., 2013 (LH)].

**FIGURE 8:**
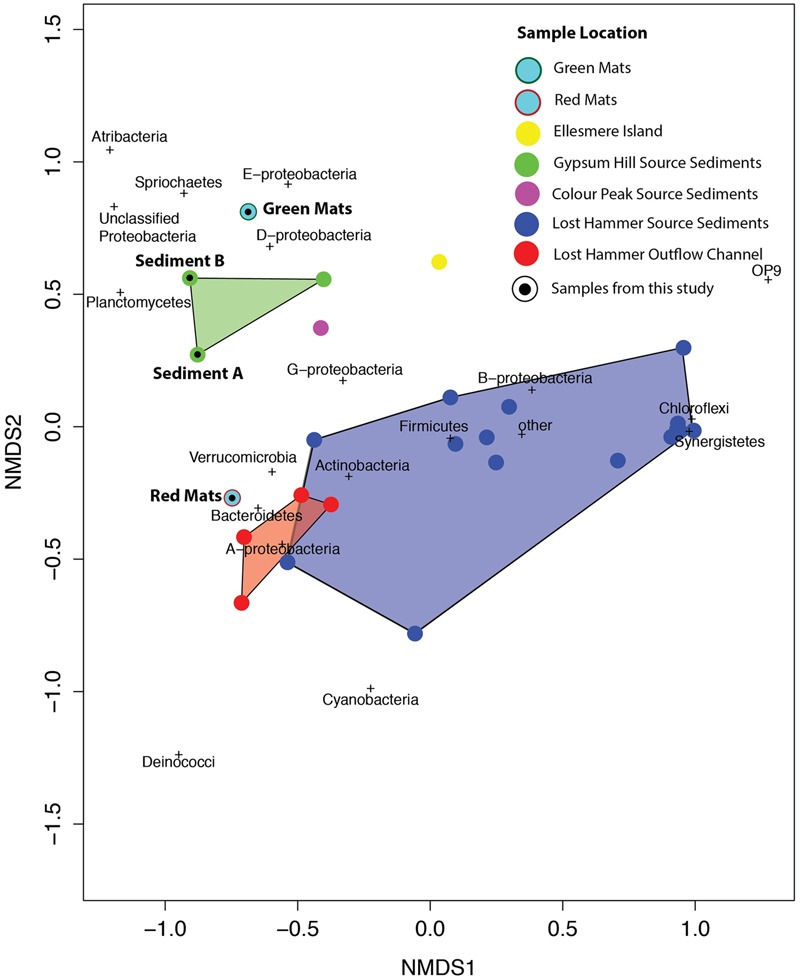
NMDS plot using the Bray–Curtis dissimilarity matrix of bacterial communities in high Arctic environments. Bacterial communities cluster by site. The mat communities studies here do not tightly cluster with any of the previously studied communities.

In all samples from all locations, the dominant phyla include Bacteroidetes, Chloroflexi, and Gammaproteobacteria. Notably, when the samples are plotted on a 2 axis NMDS plot, they cluster by site, and not by sequencing technology (**Figure 8**). It is also notable that the two mat samples in this study do not cluster with any particular spring suggesting a unique bacterial community composition at the phylum level. For example, Betaproteobacteria and Bacteroidetes membership better defines the Lost Hammer clusters than the other spring sites that are better described by other Proteobacteria sub-classes and Atribacteria. The red mats cluster closer to the Lost Hammer samples than the green mat samples consistent with a higher proportion of Bacteroidetes in the red mats relative to the green mats dominated by Betaproteobactia. These distinct bacterial communities suggest that the differences in physiochemical parameters between the spring sites play a role in community structure. Metabolic roles cannot adequately be inferred through 16S rRNA targeted-amplicon sequencing studies and detailed paired studies analyzing the spring geochemistries and transciptomic analyses are required to further comment on plausible mechanisms leading to the distinct microbial assemblages observed between sites.

### Bacterial Community Structure

#### Niche Partitioning

*Marinobacter*, more abundant in the RM sample, is a ubiquitous Gram-negative anaerobic Gammaproteobacteria in marine environments known for its ability to degrade hydrocarbons including aliphatics, polycyclic aromatics and acyclic isoprenoids (Gauthier et al., 1992; Liu et al., 2012). *Marinobacter* spp. lack fermentative metabolism, have a restricted nutritional profile, and respire nitrate. It is interesting to note that *Psychroflexus*, also more abundant in the RM sample, is also capable of nitrate reduction. However, a *Marinobacter sp.* isolated from a cold spring in Borup Fjord was found to be a key sulfide oxidizer, and the first known *Marinobacter* isolate known to biomineralize sulfur. Additionally, the soxB gene for sulfur oxidation was present in Marinobacter previously isolated from GH (Perreault et al., 2008) If the *Marinobacter sp.* in RM is capable of sulfide oxidation, *Thiomicrospira* and *Marinobacter* may be competing for resources and competition could partially explain spatial separation. More work isolating and identifying the dominant *Marinobacter* in RM is required to show sulfide oxidation, if so, the metabolic diversity is consistent with niche partitioning based on resource competition.

Nitrate metabolism could also explain the difference in community structure between the red and green mats, although more detailed studies directed at the metabolic potential and activity of the communities is required. The electron microscopy results suggest that the RM communities are more intimately associated with the physical substrate. It is not clear if the difference in community structure also represents different stages in colonization such as if the development of cohesive mats associated with sulfate reducing bacteria precedes or follows a substrate-associated, sulfide oxidizing community, or if the structure is representative of geochemical heterogeneous distribution of key nutrient and energy sources.

#### Microbial Source

The source of the microbial communities in the AHI springs remains enigmatic. The dependence on chemolithoautotrophy in the springs and similarity with subsurface and hydrothermal vent communities suggests a subsurface source. The water source is similarly unconstrained. Here we show that there is a difference in the dominant bacterial members between the Gypsum Hill source pool as samples by the Gypsum Hill sediments and surface mats in an associated flood plain suggesting fundamentally different sources. However, deeper sampling including both increased physical and technological replicates is necessary to assess the significance of these observed differences. Dispersal, transport of viable cells over geographic distances resulting in the colonization of new habitats, is one of the most important processes defining the microbial biogeography of cold environments (Margesin and Miteva, 2011). Given the seasonal habitability of the mats, the distinct geochemical parameters and the geographic relationship to the GH outflow, whether the structure of the core bacterial community is a result of endemic or cosmopolitan distribution is still unclear.

### Habitat Considerations

Here we show the presence of a microbial niche in summer surface microbial mats associated with flood plains likely fed by Gypsum Hill runoff channels. The microbial composition of these mats is unique with respect to other bacterial communities reported from other Arctic springs on AHI (e.g., Perreault et al., 2007, 2008; Niederberger et al., 2010; Lay et al., 2013; Lamarche-Gagnon et al., 2015). We suggest that seasonal shift produces ephemeral surface habitats that are not populated by through spring runoff channels. The fact that the surface is only habitable in the summer months suggests seasonality to the activity of these communities.

While results from this study are hint at a surficial community active only during the summer months, future metagenomics and metatranscriptomic analyses would lead to a better understanding of the metabolic potential and active members inhabiting these mat communities. A deeper assessment of the mat cyanobacterial diversity would allow for a more thorough comparison to other mat communities and replicate analyses are required to better understand seasonal variability and stability.

At present the surface conditions on Mars are inconsistent with the formation of standing bodies of water. The subsurface represents the most temporally extensive habitable and potentially inhabited environment on Mars. Water ice is present in the near Martian subsurface (10 s of cm; Bandfield, 2007) with polar layer deposits upwards of 1 km thick (Picardi et al., 2005). Recent radar observations have demonstrated the existence of large volumes of ground ice in the near surface environment (Stuurman et al., 2016). Modern subsurface water may be present in pockets due to radiogenic heating and lithostatic pressure and the presence of brines depressing the freezing point (Clifford et al., 2010); droplets of salts on the Phoenix lander legs demonstrate the thermodynamic stability of brines on Mars (Rennó et al., 2009). Both recurring slope lineae and (Ojha et al., 2015) Martian slope streaks (Bhardwaj et al., 2017) have been hypothesized as indicators of trainset water activity on the modern Martian surface. If these features represent surface expressions of subsurface briny aquifers (e.g., Stillman et al., 2016), transient liquid water on the surface could potentially transport subsurface microbial communities, or evidence of these communist to the surface. Alternatively, RSL features may be formed in the absence of flowing liquid water through phenomena such as deliquescence (Heinz et al., 2016). The presence of salts depresses the freezing point of water raising the depth at which liquid water could theoretically be stable. However, the presence of salt can also lower the water activity below that required for life (Tosca et al., 2008). The absence of sulfates and chlorates associated with clay-rich terrains on Mars suggests that liquid water was not always characterized by extreme salinity indicating potentially more habitable conditions in the past.

On Axel Heiberg Island, the perennial cold spring such as Gypsum Hill represent the surface expression of subsurface briny aquifers flowing through thick permafrost. Our results here suggest that the communities within these springs are distinct from the transient surficial communities that form in flood plains during the summer months. Detailed sampling over the course of several consecutive seasons is required to better understand the variability in the surface communities and their persistence through the winter. Understanding the geomicrobiology and microbial activity in hypersaline cold springs like Gypsum Hill shed insight into the potential habitability of putative subsurface Martian brines. The development of the mat communities demonstrates the potential for ephemeral environments linked to seasonal melt events, and/or flooding to host diverse and unique microbial communities.

## Conclusion

The bacterial diversity, presence of diatoms, cyanobacteria, and coloration described for the first time in the surficial mats associated with the Gypsum Hill summer flood plain is distinct from the source pool. This provides support for the hypothesis that the communities in the Gypsum Hill spring are sourced from the subsurface based on diversity differences between surficial communities as estimated by the 16s rRNA profiling. The mat communities are distinctly different from both the spring outlet and the snow-covered winter streamers suggesting temporal and spatial heterogeneity in community composition. The red mat bacterial community in particular is the first microbial community to be isolated from hypersaline cold spring systems that are not dominated by *Thiomicrospira*, but rather by *Marinobacter* (40.6%). There is a need for studies characterizing extreme environments such as the hypersaline cold springs in the Canadian High Arctic to focus on physical replicates.

We have been able to link macro-scale visual differences to distinct microbial communities that show very different micro-scale cell-substrate associations. The microbial communities of the red and green mats differ also from the reported microbial communities of the Gypsum Hill outlet (Perreault et al., 2007). Our data suggests that minor variations in chemistry, even between propinquitous sites, can have significant implications for community structure. Detailed studies of emerging, transiently habitable niches in Mars analog environments will aid our understanding of the potential for microbial life, and its detection, in putatively habitable environments beyond Earth.

## Author Contributions

HS prepared the manuscript, isolated the nucleic acids, performed the analyses, electron microscopy, and data reduction. JR greatly assisted with the manuscript preparation, data analyses, and laboratory work. RC, IR-B, and JR conducted the fieldwork, collected the samples and environmental metadata and participated in manuscript and data discussions. GO contributed to the discussion, and made possible the electron microscopy. LW conceived the design, organized the field expeditions, provided all laboratory space and materials, and contributed to the manuscript preparation and discussion.

## Conflict of Interest Statement

The authors declare that the research was conducted in the absence of any commercial or financial relationships that could be construed as a potential conflict of interest.
