# Supplementary material for: Biological Characterization of Microenvironments in a Hypersaline Cold Spring Mars Analog
Source: Front Microbiol. 2017 Dec 22;8:2527. doi: 10.3389/fmicb.2017.02527 (PMC5744183; doi:10.3389/fmicb.2017.02527)
Supplement: Supplementary file 1 [file Data_Sheet_1.docx]

Supplementary Material

Biological Characterization of Microenvironments in a Hypersaline Cold Spring Mars Analog

Haley M. Sapers*, Jennifer Ronholm, Isabelle Raymond-Bouchard, Raven Comrey, Gordon R. Osinski, Lyle G. Whyte

*** Correspondence:** Corresponding Author: haley.sapers@gmail.com

# Supplementary Table S1: Table S1: Data sources for bacterial diversity comparisons between Arctic spring communities

| sample # | collection site | sample depth | Collection year | season | method | database |
| --- | --- | --- | --- | --- | --- | --- |
|  |  |  |  |  |  |  |
| 1 | Lost Hammer outlet | NA | 2005-2006 | summer | 16S rRNA gene clone libraries | RDP II 2007 |
| 2 | Lost Hammer outflow | shallow | 2007-2008 | winter | 16S rRNA gene clone libraries | RDP II 2003 |
| 3 | Lost Hammer outflow | shallow | 2008 | winter | 16S rRNA gene clone libraries | RDP II 2003 |
| 4 | Lost Hammer outflow | shallow | 2008 | winter | 16S rRNA gene clone libraries | RDP II 2003 |
| 5 | Lost Hammer outflow | shallow | 2007 | winter | 16S rRNA gene clone libraries | RDP II 2003 |
| 6 | Lost Hammer outlet | shallow | 2009 | summer | metagenomic, Roche 454 GS FLX Titanium | MG-RAST |
| 7 | Lost Hammer outlet | shallow | 2010 | summer | cDNA, Roche 454 GS FLX Titanium | GenBank nr |
| 8 | Lost Hammer outlet | shallow | 2012 | winter | cDNA Roche 454 GS FLX Titanium | GreenGenes 2006 |
| 9 | Lost Hammer outlet | shallow | 2012 | winter | DNA Roche 454 GS FLX Titanium | GreenGenes 2006 |
| 10 | Lost Hammer outlet | middle | 2012 | winter | cDNA Roche 454 GS FLX Titanium | GreenGenes 2006 |
| 11 | Lost Hammer outlet | middle | 2012 | winter | DNA Roche 454 GS FLX Titanium | GreenGenes 2006 |
| 12 | Lost Hammer outlet | deep | 2012 | winter | cDNA Roche 454 GS FLX Titanium | GreenGenes 2006 |
| 13 | Lost Hammer outlet | deep | 2012 | winter | DNA Roche 454 GS FLX Titanium | GreenGenes 2006 |
| 14 | Lost Hammer outlet | shallow | 2012 | summer | cDNA Roche 454 GS FLX Titanium | GreenGenes 2006 |
| 15 | Lost Hammer outlet | shallow | 2012 | summer | DNA Roche 454 GS FLX Titanium | GreenGenes 2006 |
| 16 | Lost Hammer outlet | middle | 2012 | summer | cDNA Roche 454 GS FLX Titanium | GreenGenes 2006 |
| 17 | Lost Hammer outlet | middle | 2012 | summer | DNA Roche 454 GS FLX Titanium | GreenGenes 2006 |
| 18 | Lost Hammer outlet | deep | 2012 | summer | cDNA Roche 454 GS FLX Titanium | GreenGenes 2006 |
| 19 | Lost Hammer outlet | deep | 2012 | summer | DNA Roche 454 GS FLX Titanium | GreenGenes 2006 |
| 20 | Gypsum Hill outlet | shallow | 2004 | summer | 16S rRNA gene clone libraries | GenBank |
| 21 | Gypsum Hill outlet | shallow | 2014 | summer | DNA Roche 454 GS FLX Titanium | Silva v124 |
| 22 | Gypsum Hill outlet | shallow | 2014 | summer | DNA Roche 454 GS FLX Titanium | Silva v124 |
| 23 | Gypsum Hill outflow | shallow | 2013 | summer | DNA Roche 454 GS FLX Titanium | Silva v124 |
| 24 | Gypsum Hill outflow | shallow | 2013 | summer | DNA Roche 454 GS FLX Titanium | Silva v124 |
| 25 | Colour Peak outlet | shallow | 2004 | summer | 16S rRNA gene clone libraries | GenBank |
| 26 | Ellesmere Island | NA | 2006 | summer | 16S rRNA gene clone libraries | GreenGenes 2006 |

^1^ Niederberger, T. D., Perreault, N., Tille, S., Lollar, B. S., Lacrampe-Couloume, G., Andersen, D. T., et al. (2010). Microbial characterization of a subzero, hypersaline methane seep in the Canadian High Arctic. ISME J. 4, 1326–1339. doi: 10.1038/ismej.2010.57

^2^Lay, C.-Y., Mykytczuk, N. C. S., Niederberger, T. D., Martineau, C., Greer, C. W., and Whyte, L. G. (2012). Microbial diversity and activity in hypersaline high Arctic spring channels. Extremophiles 16, 177–191. doi: 10.1007/s00792-011- 0417-9

^3^Lay, C.-Y., Mykytczuk, N. C. S., Yergeau, E., Lamarche-Gagnon, G., Greer, C. W., and Whyte, L. G. (2013). Defining the functional potential and active community members of a sediment microbial community in a higharctic hypersaline subzero spring. Appl. Environ. Microbiol. 79, 3637–3648. doi: 10.1128/AEM.00153-13

^4^Lamarche-Gagnon, G., Comery, R., Greer, C. W., and Whyte, L. G. (2015). Evidence of in situ microbial activity and sulphidogenesis in perennially sub-0 C and hypersaline sediments of a high Arctic permafrost spring. Extremophiles 19, 1–15. doi: 10.1007/s00792-014-0703-4

^5^Perreault, N., Andersen, D. T., Pollard, W. H., Greer, C. W., and Whyte, L. G. (2007). Characterization of the prokaryotic diversity in cold saline perennial springs of the Canadian high Arctic. Appl. Environ. Microbiol. 73, 1532–1543. doi: 10.1128/AEM.01729-06

^6^This study

^7^Gleeson, D. E., Williamson, C., Grasby, S. E., Pappalardo, R. T., Spear, J. R., and Templeton, A. S. (2011). Low Temperature S0 biomineralization at a supraglacial spring system in the Canadian High Arctic. Geobiology 9, 360–375. doi: 10.1111/j.1472-4669.2011.00283.x
